# Integrating GEO Database, Mendelian Randomization, and Molecular Docking to Identify HLA-C as a Potential Therapeutic Target for Periodontitis

**DOI:** 10.1155/mi/9163431

**Published:** 2025-07-14

**Authors:** ChengJi Shi, Xinyi Ou, LiJuan Huang, XiaoXu Lei, ShuHao Xu, Menglu Ou, Wei Li, Xi Zhao

**Affiliations:** ^1^Department of Stomatology, The People's Hospital of Deyang City, Deyang, Sichuan, China; ^2^Nanobiosensing and Microfluidic Point-of-Care Testing, Key Laboratory of Luzhou, Department of Clinical Laboratory, The Affiliated Traditional Chinese Medicine Hospital, Southwest Medical University, Luzhou, Sichuan, China; ^3^School of Public Health, Nanchang Medical College, Nanchang, Jiangxi, China

**Keywords:** eQTL, GWAS, Mendelian randomization, periodontal disease, single-cell sequencing, WGCNA

## Abstract

**Background:** Periodontitis is a chronic inflammatory disease that leads to the destruction of periodontal tissues, ultimately resulting in tooth loss. Current treatments primarily focus on mitigating inflammation and alleviating symptoms, but they often lack specificity. This study aims to explore the molecular mechanisms of periodontitis using gene expression databases (GEOs) and bioinformatics methods, combined with Mendelian randomization (MR) analysis, to identify key therapeutic targets.

**Methods:** This study analyzed GSE10334 microarray data to identify differentially expressed genes (DEGs) in periodontitis using R. Weighted gene co-expression network analysis (WGCNA) identified key gene modules and enrichment analysis revealed functional pathways. Immune infiltration was assessed with CIBERSORT and MR explored human leukocyte antigen C's (HLA-C's) role. Single-cell analysis using Seurat identified cell types and CellChat mapped cell communication. Molecular docking (MD) and molecular dynamic simulations were used to validate the interaction between the hub target genes and the potential drug.

**Results:** Differential expression analysis identified 167 DEGs in periodontitis. WGCNA revealed a strong association with the blue module. MR analysis confirmed HLA-C as a risk factor. Single-cell RNA sequencing (scRNA-seq) showed elevated plasmablasts and HLA-C expression. MD and molecular dynamic simulation analysis identified metronidazole as a potential drug with stable binding to HLA-C, forming a stable complex with no significant conformational changes during the 100 ns simulation period.

**Conclusion:** This study identifies HLA-C as a potential therapeutic target for periodontitis, with MD studies and molecular dynamic simulations highlighting metronidazole as a potential treatment. These findings provide new insights into periodontitis pathogenesis and potential therapeutic strategies.

## 1. Introduction

Periodontitis is a chronic inflammatory disease triggered by the host immune response to commensal and pathogenic oral bacteria, leading to the gradual destruction of periodontal tissues [1]. As one of the most prevalent oral diseases, it has a profound impact on global oral health and patients' quality of life [2]. Diagnosis of periodontitis is primarily based on clinical manifestations such as the formation of periodontal pockets, gingival inflammation, and alveolar bone resorption, augmented by advanced diagnostic techniques, including radiographic imaging and biomarker profiling [3–5]. The application of biomarkers, such as inflammatory cytokines and enzymes, has greatly enhanced the diagnostic efficiency and monitoring efficiency of periodontitis [4, 6]. Early, timely, and accurate diagnosis, along with effective evidence-based treatment measures, is crucial for improving the prognosis of periodontitis patients. Therefore, the exploration of more specific biomarkers related to periodontitis has become an urgent priority.

Due to the rapid progress in bioinformatics and microarray technologies, studies on periodontitis have increasingly employed more integrative bioinformatics approaches [7]. Previously, researchers analyzed periodontitis patient samples using microarray technology to identify key genes associated with the disease [8]. Weighted gene co-expression network analysis (WGCNA), a systems biology approach, has gained widespread use in elucidating the network relationships between genes and molecular pathways, particularly in the identification of potential therapeutic targets and biomarkers [9, 10]. By clustering genes with similar expression patterns into co-expression modules, WGCNA effectively reveals the correlations between sample traits and gene modules [11, 12]. This method not only identifies key genes related to the disease but also reveals the interaction networks between genes, helping us gain a deeper understanding of the molecular mechanisms of periodontitis [13, 14]. By combining single-cell RNA-sequencing (scRNA-seq) technology, researchers can obtain more precise information on cell types and states, providing stronger support for the accurate diagnosis and treatment of periodontitis [15, 16]. The integration of WGCNA and scRNA-seq fosters novel research directions and expedites the identification of potential biomarkers and therapeutic targets.

Mendelian randomization (MR) is an effective analytical approach that leverages genetic variation to evaluate whether the observed association between risk factors and outcomes reflects a causal relationship [17, 18]. Its theoretical foundation lies in the random distribution of genetic variants during meiosis, which results in genetic variations being randomly assigned at birth. These variations may influence the distribution of risk factors or outcomes [19]. MR employs instrumental variables (IVs) to further investigate causal relationships between samples. These IVs must satisfy the following conditions: (1) strong association with the exposure factor, (2) no direct relationship with the outcome, and (3) independence from confounding factors [20]. By addressing confounding bias and reverse causation inherent in traditional observational studies, MR provides a reliable framework for identifying causal links between genetic variants and complex traits or diseases. In the context of periodontal disease, MR analysis can integrate genetic instruments associated with potential biomarkers, thereby elucidating the genetic mechanisms underlying disease susceptibility and progression, thus, contributing valuable insights for advancing precision dentistry [21].

In this study, we identified 118 differentially expressed genes (DEGs) and core gene modules significantly associated with periodontitis through gene expression database (GEO) and WGCNA analyses. Further investigation revealed that key genes, including human leukocyte antigen C (HLA-C), STAP1, PTP4A3, NEU1, IGLV1-44, IGLL5, and NIPAL4, play critical roles in the pathogenesis of periodontitis. MR analysis demonstrated a significant positive correlation between HLA-C expression levels and periodontitis, suggesting that HLA-C may be a potential risk factor. Immune cell infiltration analysis further indicated a close association between HLA-C and plasma cell infiltration. scRNA-seq analysis confirmed these findings, showing a marked increase in plasma cell numbers, significantly upregulated HLA-C expression in plasma cells, and enhanced intercellular communication in periodontitis patients. Molecular docking (MD) studies, complemented by molecular dynamics simulations, suggest that HLA-C has the potential to bind to metronidazole, highlighting its promising role as a therapeutic target and drug candidate.

## 2. Research Methodology and Data Sources

### 2.1. Data Source

In this study, we accessed relevant microarray datasets from the database by searching for the term “periodontitis.” The dataset GSE10334 contains samples from 19 periodontitis patients and six normal [22].

### 2.2. Identification of DEGs

The GSE10334 dataset was first normalized. After preprocessing, differential expression analysis was performed using the “limma” package to identify DEGs between the periodontitis and control groups. The criteria for statistical significance were set at *p*  < 0.05 and |log_2_ fold change (FC)| ≥ 1. To visualize the DEG results, heatmaps and volcano plots were created using the “pheatmap” and “ggplot2” packages in R.

### 2.3. WGCNA Analysis

WGCNA is used to explore co-expression patterns and biological networks in gene expression data. First, clustering analysis was performed to remove outliers. A scale-free co-expressed gene network was constructed using the one-step function and the soft threshold (*β*) was determined with “pickSoftThreshold” to optimize the adjacency matrix. The adjacency matrix was then transformed into a topological overlap matrix (TOM) and its dissimilarity (1-TOM) calculated. Modules were detected using hierarchical clustering and dynamic tree cutting. Finally, gene significance and module membership were computed to identify clinically relevant modules and the feature gene network was visualized.

### 2.4. Enrichment Analysis

After intersecting the genes from the WGCNA blue module with the DEGs, potential hub genes related to the mechanism of periodontitis were identified. These potential hub genes were subjected to Gene Ontology (GO) functional analysis and Kyoto Encyclopedia of Genes and Genomes (KEGG) pathway enrichment analysis using the “clusterProfiler” package [23]. The significant threshold for enrichment was set at *p*  < 005.

### 2.5. Immune Cell Infiltration Analysis

To assess immune cell infiltration in gene expression profiles associated with periodontitis, we utilized the CIBERSORT algorithm. This approach calculates the relative proportions of 22 immune cell subtypes (LM22) in each sample, thereby providing a comprehensive evaluation of immune cell infiltration levels.

### 2.6. MR

**Figure 1:**
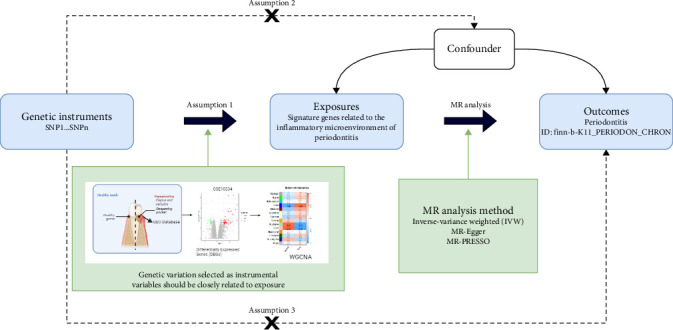
Three assumptions underpin the Mendelian randomization study: Assumption 1 is the association assumption, which represents a strong association between the instrumental variable and the exposure. Assumption 2 is the independence assumption, which states that the instrumental variable is independent of the confounders. Assumption 3 is the exclusivity assumption, which states that the instrumental variable can only affect the outcome through the exposure.

To ensure the robustness and reliability of the findings, we followed the guidelines of the STROBE-MR checklist in conducting our MR analysis [24]. The MR analysis was performed using the TwoSampleMR package. Genome-wide significant single nucleotide polymorphisms (SNPs) with *p* < 5 × 10^−8^ were selected as IVs, excluding those associated with potential confounders. Linkage disequilibrium (LD) pruning (*R*^2^ < 0.01, 10,000 kb window) ensured IV independence. Exposure data were sourced from expression quantitative trait loci (eQTLs) in the IEU Open genome-wide association study (GWAS) database (https://gwas.mrcieu.ac.uk/datasets/). Additionally, we accessed the periodontal disease cohort from this database, identified by the ID finn-b-K11_PERIODON_CHRON, which included 195,395 standard samples and 3046 samples diagnosed with periodontal disease. These samples are of European descent and provided whole genome data that served as our outcome data. The inverse variance weighted (IVW) method with a significance threshold of *p*  < 0.05 was used as the primary analysis. Subsequently, we computed the *F*-statistic to assess the strength of the association between IVs and exposure factors. SNPs with an *F*-value less than 10 were excluded to mitigate potential weak instrument bias. Cochran's *Q* test assessed heterogeneity (*p*  < 0.05 indicating potential invalid instruments), while leave-one-out analysis validated robustness. MR-Egger regression tested for directional pleiotropy (intercept *p*  < 0.05 suggesting pleiotropic bias). Additionally, we employed the MR-PRESSO package to detect and correct for horizontal pleiotropy, thereby ensuring the robustness of the results [25]. This integrated approach combined multiomics evidence to validate causal relationships between biomarkers and periodontitis. To visualize the design of this study more, we drew the flow chart (Figure 1).

### 2.7. Dimensionality Reduction Analysis of Single-Cell Data and Identification of Cell Types

Data from the GSE152042 dataset underwent quality control, normalization, and dimensionality reduction using Seurat and MonacoImmuneData frameworks. The PercentageFeatureSet function calculated the percentage of molecules and rRNA, ensuring cells expressed >500 genes, <4000 genes, with <30% molecule content and at least 100 unique molecular identifiers (UMIs) per cell. Data normalization and identification of highly variable genes were performed using FindVariableFeatures. Cells were clustered (resolution = 0.1, dim = 30) using FindNeighbors and FindClusters, and visualized with *t*-SNE. Intercellular communication was inferred, analyzed, and visualized using the “CellChat” package in R, which constructs a neighborhood graph and estimates communication probabilities based on gene expression, signal ligands, receptors, and related factors. Cell type annotation was performed using the SingleR algorithm. The SingleR algorithm classifies each cell population based on known marker genes. To validate the annotations, manual annotation was also performed, relying on well-established cell-type marker genes (Supporting Information 1: Table S1). To further validate the accuracy of the annotations, a comparison was made with the PanglaoDB database, a comprehensive resource for human and mouse scRNA-seq data (Supporting Information 2: Table S2). During the comparison, marker genes and cell-type classifications were cross-checked to ensure the accuracy of our annotations.

### 2.8. Candidate Drug Prediction

Evaluating the interactions between proteins and drugs plays a crucial role in determining whether target genes can serve as potential drug targets. In this study, the drug signatures database will be utilized for drug prediction [26]. This database contains 22,527 gene sets and 17,389 distinct compounds spanning 19,531 genes, aiming to link drugs and chemicals to their corresponding target genes. By uploading the identified target genes to DSigDB, potential drug candidates can be predicted to assess the drug activity of these target genes.

### 2.9. MD

This study utilizes MD to evaluate the binding energy and interaction patterns between drug candidates and target proteins at the atomic level, exploring their potential as therapeutic targets. The two-dimensional structures of small-molecule ligand drugs were obtained from the PubChem database (PDB, https://www.rcsb.org/), while the three-dimensional structures of target proteins (receptors) were retrieved from the RCSB Protein Data Bank (PDB, https://www.rcsb.org/). AutodockVina 1.2.2 was used to perform docking on selected drugs and their corresponding target proteins.

### 2.10. Molecular Dynamics Simulation

Molecular dynamics simulations were performed using Gromacs 2022.3. For preprocessing of small molecules, AmberTools 22 was utilized to assign the GAFF force field, while Gaussian 16W was employed to hydrogenate the molecules and compute the RESP charge. The resulting potential data were incorporated into the system's topology file. The simulation was conducted at a fixed temperature of 300 K and atmospheric pressure (1 bar). Amber99sb-ildn was selected as the force field, with Tip3p water model used as the solvent. The system's total charge was neutralized by adding appropriate Na^+^ ions. Energy minimization was performed using the steepest descent method, followed by an NVT (isothermal and isovolumic) and NPT (isothermal and isobaric) equilibration for 100,000 steps each, with a coupling constant of 0.1 ps and a duration of 100 ps. The final step involved a production simulation with a total of 50,000,000 steps, each with a time step of 2 fs, for a total simulation time of 100 ns. Upon completion of the simulation, trajectory analysis was carried out using the built-in tools in the software. Metrics such as the root-mean-square deviation (RMSD), root-mean-square fluctuation (RMSF), solvent-accessible surface area (SASA), and the radius of gyration of the protein were calculated, alongside free energy calculations (MMGBSA), free energy landscapes, and other relevant data [27].

## 3. Results

**Figure 2:**
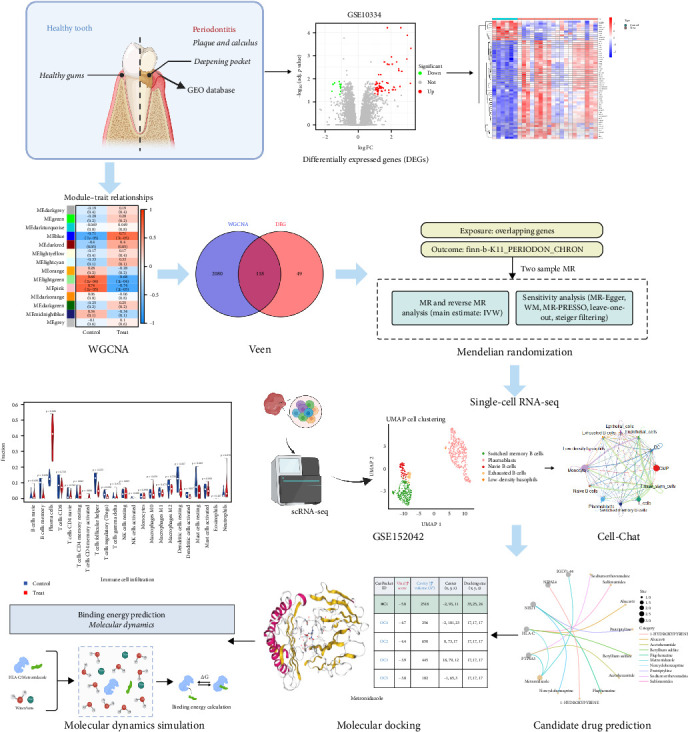
Study design overview.

The workflow is visualized in Figure 2.

### 3.1. Identification of DEGs

**Figure 3:**
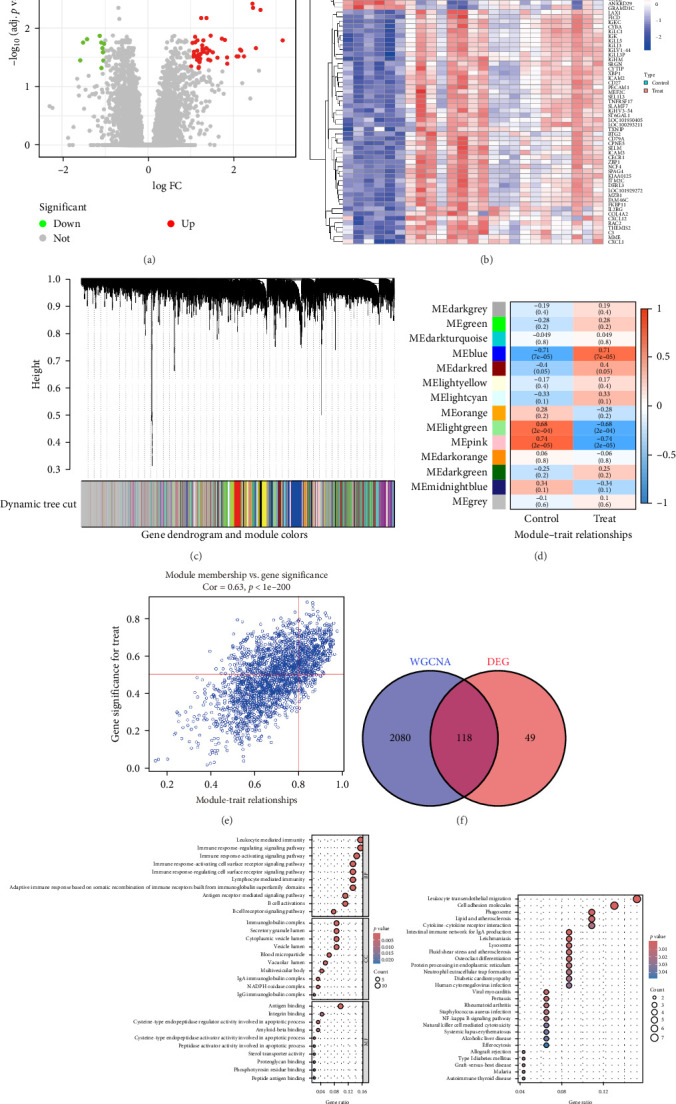
Identification and functional analysis of differentially expressed and core genes in periodontitis. (A) Volcano plot for differential expression analysis of GSE10334. (B) Heat map of differential expression analysis of GSE10334 showing 167 DEGs, with 104 upregulated and 63 downregulated. (C) A hierarchical clustering dendrogram of all genes in the GSE10334 dataset was generated based on the topological overlap matrix (1-TOM). (D) WGCNA module trait heatmap showing the correlation between clustered gene modules in the GSE10334 dataset and periodontitis. Each module includes the corresponding correlation coefficient and *p*-value. (E) In the GSE10334 dataset, the blue scatter plot of this module shows the most significant positive correlation with periodontitis. (F) Venn diagram showing 118 overlapping intersection genes. (G) GO enrichment analysis of the enriched genes. (H) KEGG enrichment analysis of the enriched genes.

We retrieved the periodontitis dataset (GSE10334) from the GEO database and performed differential expression analysis to identify DEGs in the periodontitis group. Compared to the normal group, we found 167 DEGs in the periodontitis group, with 104 upregulated and 63 downregulated (Figure 3A,B).

### 3.2. Construction of WGCNA Network

We then conducted WGCNA on the core genes from the GSE10334 dataset to explore gene expression patterns related to periodontitis (Figure 3C). Fourteen distinct modules were identified (Figure 3D). Correlation analysis revealed that the blue module exhibited the strongest association with periodontitis (Figure 3E).

### 3.3. Enrichment Analysis

We identified disease-related core genes and overlapping genes between DEGs using the WGCNA method. Ultimately, we selected 118 overlapping genes as potential core genes involved in the development and progression of periodontitis (Figure 3F). Next, we carried out GO and KEGG enrichment analyses to examine the biological roles of these genes. The results of the GO analysis revealed that the overlapping genes are closely related to several immune-related biological processes, including immune response-activating cell surface receptor signaling, leukocyte-mediated immunity, B cell receptor signaling, immune response regulation, and B cell activation (Figure 3G). The KEGG analysis indicated that these candidate genes are primarily involved in immune system functions, signaling molecule interactions, parasitic infectious diseases, and transport and metabolic pathways (Figure 3H).

### 3.4. Immune Cell Infiltration

**Figure 4:**
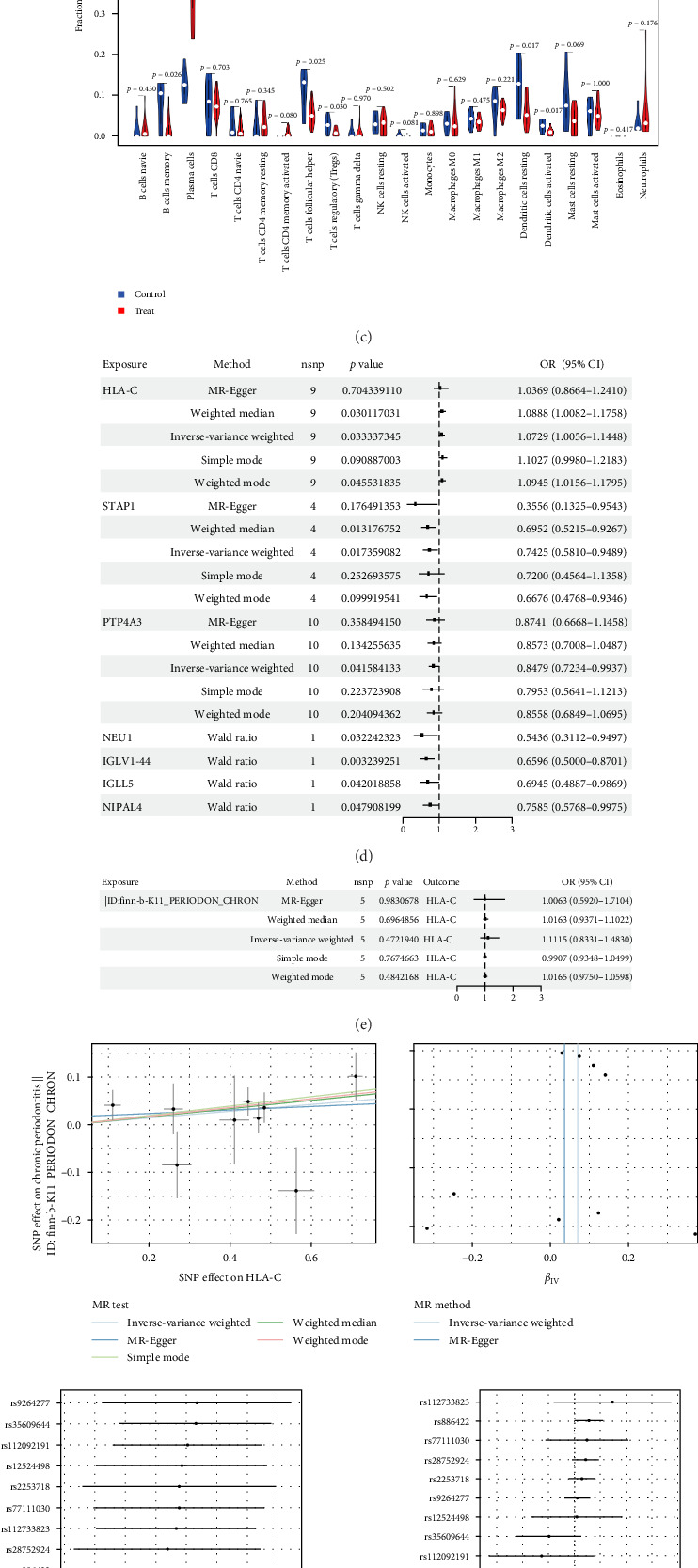
Immune cell infiltration and MR analysis results. (A, B) Immune cell infiltration analysis using the CIBERSORT algorithm comparing the periodontitis and control groups, showing the relative distribution of 22 immune cell types in all periodontitis samples. (C) Immune cell infiltration analysis using the CIBERSORT algorithm showing differences in the distribution of 22 immune cell types between periodontitis and control groups. (D) MR forest plot of enriched genes and periodontitis. (E) Validation of the reverse MR analysis between periodontal disease and HLA-C. (F) The scatter plot, forest plot, funnel plot, and leave-one-out plot display the causal effect of HLA-C on periodontal disease risk and its heterogeneity. (G) The difference in immune cell infiltration between periodontal disease and controls. (H) The correlation between HLA-C and infiltrating immune cells in periodontal disease.

The CIBERSORT algorithm was employed to analyze immune cell infiltration in both the periodontitis and control groups (Figure 4A,B). The results showed significant differences between the two groups. In both groups, the infiltration of immune cells such as plasma cells, memory B cells, CD4^+^ memory T cells, activated T cells, follicular helper T cells, regulatory T cells (Tregs), resting dendritic cells (DCs), and activated DCs showed marked differences (Figure 4C). These results suggest a close relationship between immune cell infiltration and gene expression differences.

### 3.5. There Is a Causal Relationship Between HLA-C and the Risk of Periodontitis

The eQTL data were sourced from the IEU OpenGWAS project and periodontitis GWAS data were obtained from FinnGen R11. To investigate the genetic relationship between 118 potential genes and periodontal disease, we conducted a combined analysis of GWAS and MR, identifying seven core genes. Among them, HLA-C (IVW, nine SNPs, *p*=0.03) was identified as a risk factor for periodontitis, while STAP1 (IVW, four SNPs, *p*=0.01) and PTP4A3 (IVW, 10 SNPs, *p*=0.04) were recognized as protective factors (Figure 4D).

We then further investigated the causal relationship between HLA-C and periodontitis. Using the IVW method, a significant association was observed, with an odds ratio (OR) of 1.0729 (95% CI: 1.0056–1.1448, *p*=0.033). The MR-Egger test revealed no evidence of heterogeneity (*p*=0.704; Figure 4D). Moreover, reverse MR analysis showed no evidence of reverse causality, suggesting that HLA-C is not inversely related to periodontitis (Figure 4E). The MR-Egger regression intercept also indicated no pleiotropy (*p*=0.704), further supporting the robustness of our findings. Sensitivity analysis using the leave-one-out approach demonstrated that no individual polymorphism significantly influenced the results, confirming the reliability of the causal effect estimates (Figure 4F).

Additionally, correlation analysis with 22 types of immune cells revealed that HLA-C was positively correlated with plasma cells (*r* = 0.59), while it was negatively correlated with resting DCs (*r* = −0.50) and CD8^+^ T cells (*r* = −0.53; Figure 4G,H). These results suggest a close relationship between immune cell infiltration and gene expression differences.

### 3.6. Analysis of Single-Cell RNA Sequencing Data of Marker Gene

**Figure 5:**
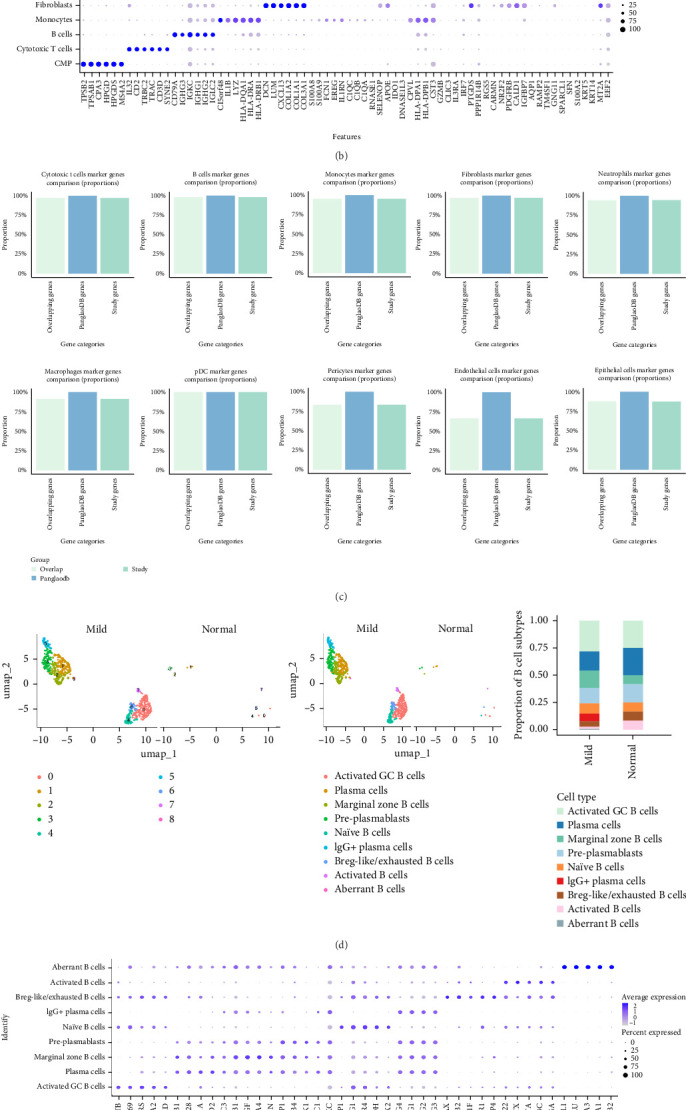
Single-cell transcriptomic analysis of normal controls and periodontitis patients. (A) UMAP cell clustering of all samples in the single-cell dataset, with different colors representing distinct cell clusters. (B) Expression levels of marker genes for each cell cluster. (C) The comparison with the PanglaoDB database showed strong consistency between our annotations and those in the database, particularly in terms of key marker genes and cell-type classifications. (D) Distribution of B cells in periodontal disease and control groups. (E) Expression levels of marker genes for each cell cluster. (F) The comparison with the PanglaoDB database indicates that our annotation of plasmablasts is highly consistent with the classification in the database.

**Figure 6:**
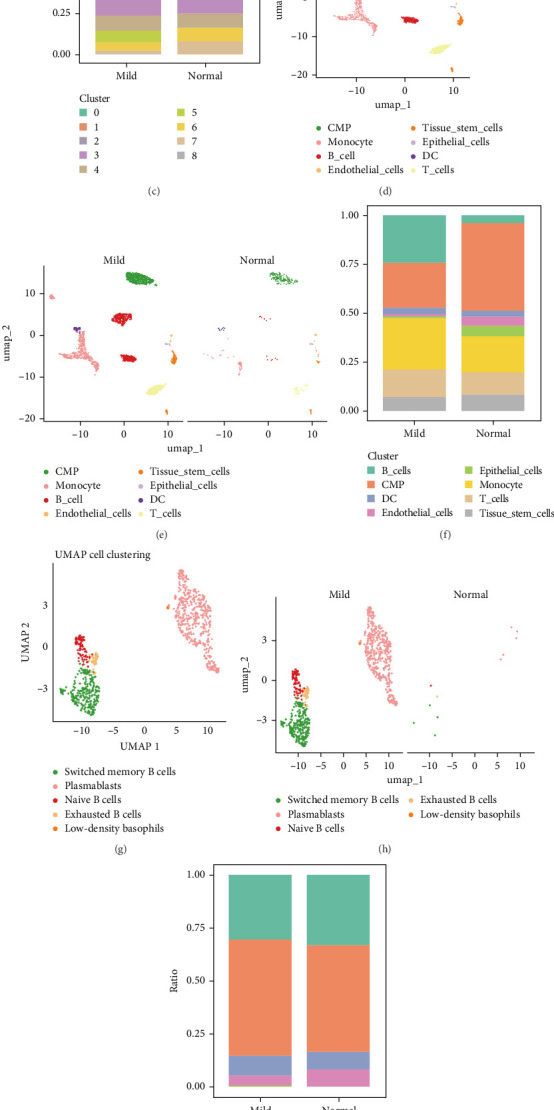
Single-cell transcriptomic analysis of normal controls and periodontitis patients. (A) UMAP cell clustering of all samples in the single-cell dataset, with different colors representing distinct cell clusters. (B) Distribution of cell clusters in periodontal disease and control samples. (C) Percentage of different cell types in both periodontal disease and control groups. (D, E) Distribution of various cell types in periodontal disease and control groups. (F) Percentage of different cell types in both periodontal disease and control groups. (G, H) Distribution of B cells in periodontal disease and control groups. (I) Percentage of different B cell types in periodontal disease and control groups. (J) Expression of HLA-C in B cells from normal controls and periodontitis samples.

The annotations from SingleR were largely consistent with the manual annotations, particularly for B cell populations, where the clustering results were almost identical (Figure 5A,B). Minor discrepancies were observed in other cell types, such as fibroblasts and pericytes. The comparison with the PanglaoDB database showed strong consistency between our annotations and those in the database, especially in terms of key marker genes and cell-type classifications (Figure 5C). These results further reinforce the confidence in the accuracy of our cell-type annotations, particularly for B cell subsets and other major cell types (Figure 5D–F). In the GSE152042 dataset, uniform manifold approximation and projection (UMAP) clustering identified 14 distinct cell clusters (Figure 6A,B). Using SingleR, eight cell types were annotated across all samples, including CMP, monocytes, B cells, endothelial cells, tissue stem cells, epithelial cells, DCs, and T cells (Figure 6D,E). Dimensionality reduction and proportional representation analysis revealed a significant increase in the proportion of B cells in periodontal disease samples, indicating heightened B cell activity during the disease progression (Figure 6F). Further analysis of immune cell marker gene expression identified five B cell subtypes: switched memory B cells, plasmablasts, maive B cells, exhausted B cells, and low-density basophils. Corresponding dimensionality reduction and proportional representation plots were also generated. Notably, the number of plasmablasts was significantly higher in periodontal disease patients compared to healthy controls, suggesting a strong correlation between plasmablast levels and the severity of periodontal disease (Figure 6G–I). Additionally, the results showed elevated HLA-C expression in periodontal disease samples, particularly in plasmablasts (Figure 6J).

### 3.7. Comparative Analysis of Cell–Cell Interactions

**Figure 7:**
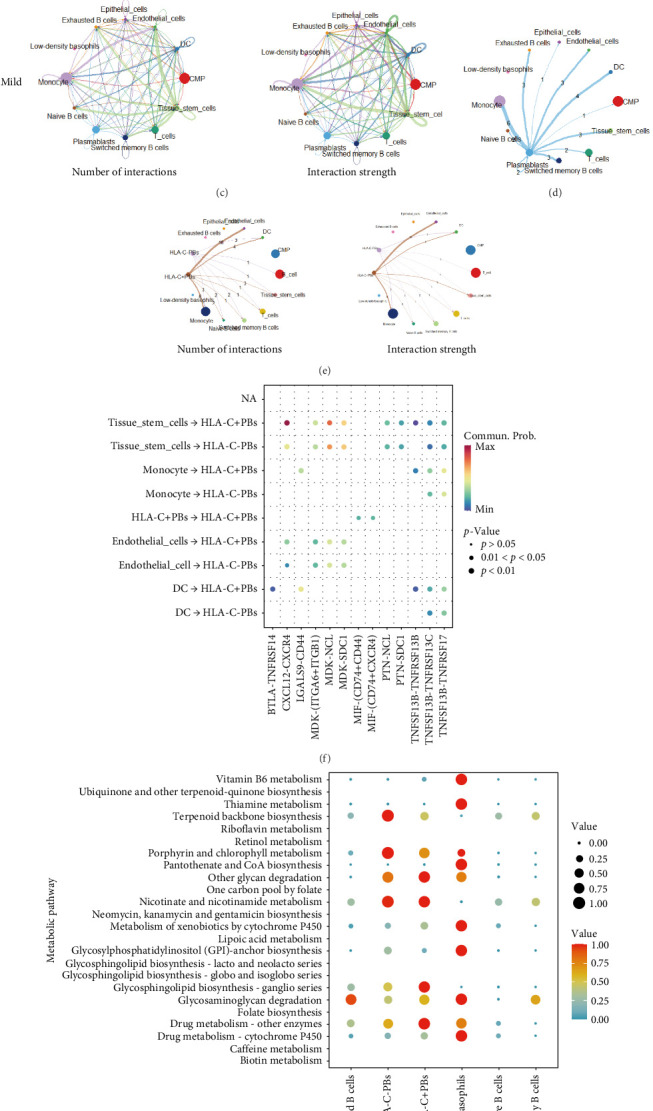
Cell–cell communication and metabolic pathways in normal controls and periodontitis. (A) Comparison of ligand–receptor interactions and communication intensity between normal control samples. (B) Cell–cell communication between plasmablasts and other cells in normal control samples. (C) Comparison of ligand–receptor interactions and communication intensity between periodontal disease samples. (D) Cell–cell communication between plasmablasts and other cells in periodontal disease samples. (E, F) Ligand–receptor interaction networks between HLA-C positive and negative plasmablasts and other cell subsets, showing the number of receptor–ligand pairs, with line thickness representing interaction strength. (G) Bubble plot of enriched metabolic pathways highlighting metabolic differences between HLA-C positive and negative plasmablasts. Each bubble represents a metabolic pathway, with *p*-values and the total number of involved metabolites listed on the right.

We compared the interaction networks of different cell subsets between normal control and periodontal disease samples. Cell communication analysis revealed that, compared to the normal controls, the periodontal disease group exhibited a higher number of ligand–receptor interactions and greater communication intensity among the eight cell types analyzed (Figure 7A–C). Notably, in the normal control group, plasmablasts showed minimal or no interactions with other cell types (Figure 7B). In contrast, in periodontal disease samples, activated B cells facilitated significant interactions between plasmablasts and various other cell types (Figure 7D). This suggests that plasmablasts may play a pivotal role in the pathogenesis of periodontal disease through crosstalk with a range of immune cells.

Subsequently, we investigated the cell–cell communication and metabolic pathways of plasmablasts between HLA-C positive and negative groups. The results showed that plasmablasts in the HLA-C positive group exhibited significantly stronger communication intensity compared to the negative group, indicating extensive interactions with various cell types (Figure 7E). Compared to the HLA-C negative Tissue_stem_cell group, the HLA-C positive group also demonstrated enhanced MDK-NCL and CXCL12-CXCR4 signaling pathways in cell communication (Figure 7F). At the metabolic level, differential expression was concentrated in pathways such as drug metabolism—other enzymes, drug metabolism—cytochrome P450, glycosaminoglycan degradation, lysosphingolipid biosynthesis—ganglio series, terpenoid backbone biosynthesis, and porphyrin and chlorophyl metabolism (Figure 7G). Notably, the HLA-C positive group showed higher expression levels and stronger correlations in the drug metabolism—other enzymes and Glycosphingolipid biosynthesis—ganglio series pathways (Figure 7G).

### 3.8. Prediction of Potential Drug Candidates and MD Analysis

**Figure 8:**
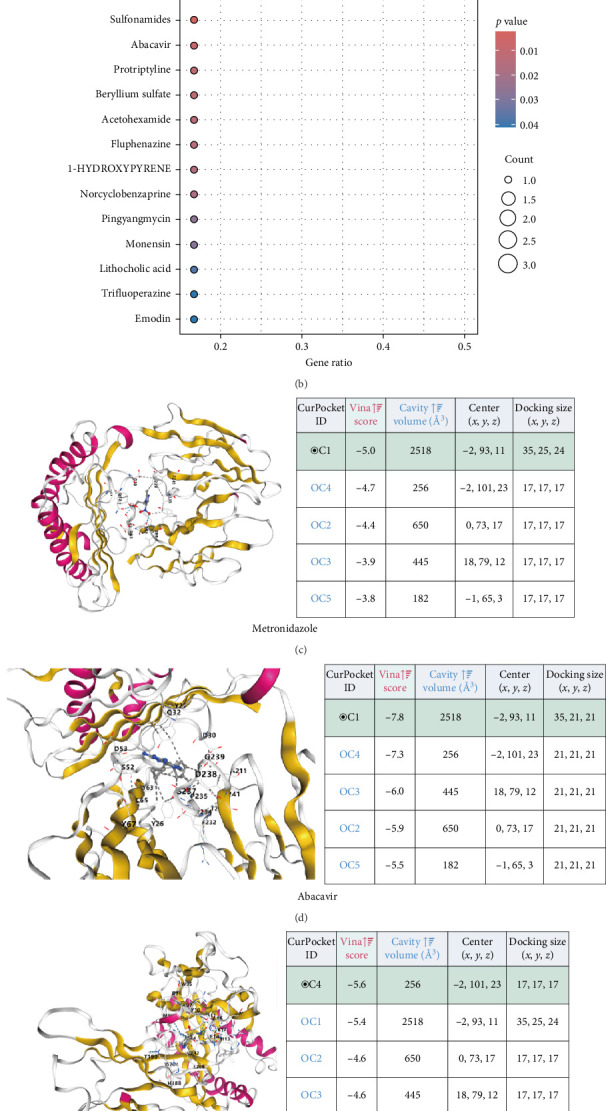
Prediction of potential therapeutic drugs and molecular docking analysis. (A) Top 15 potential compounds predicted using the DSigDB database based on adjusted *p*-values. (B) Metronidazole identified as the most significant drug associated with HLA-C. (C–F) Molecular docking analysis of four candidate drugs with potential binding sites on HLA-C, showing effective docking outcomes and interactions.

The DSigDB database was utilized in this study to predict potential therapeutic drugs. Based on adjusted *p*-values, the top 15 potential compounds were identified (Figure 8A). Among these, metronidazole (Rhein TTD 00010611/CTD 00001002) was determined to be the most significant drug associated with HLA-C (Figure 8B). To evaluate the binding affinity of the candidate drugs to their targets and assess the druggability of these targets, MD analysis was conducted.

Four candidate drugs with potential binding sites on HLA-C were identified, yielding four effective docking results (Figure 8C–F). Each candidate drug exhibited clear hydrogen bonds and strong electrostatic interactions with its protein target. Furthermore, the binding pockets of HLA-C were successfully occupied by all four candidate drugs. Notably, HLA-C and metronidazole displayed a binding energy of −5.00 kcal/mol, indicating a relatively stable interaction (Figure 8C).

### 3.9. Molecular Dynamics Simulation

**Figure 9:**
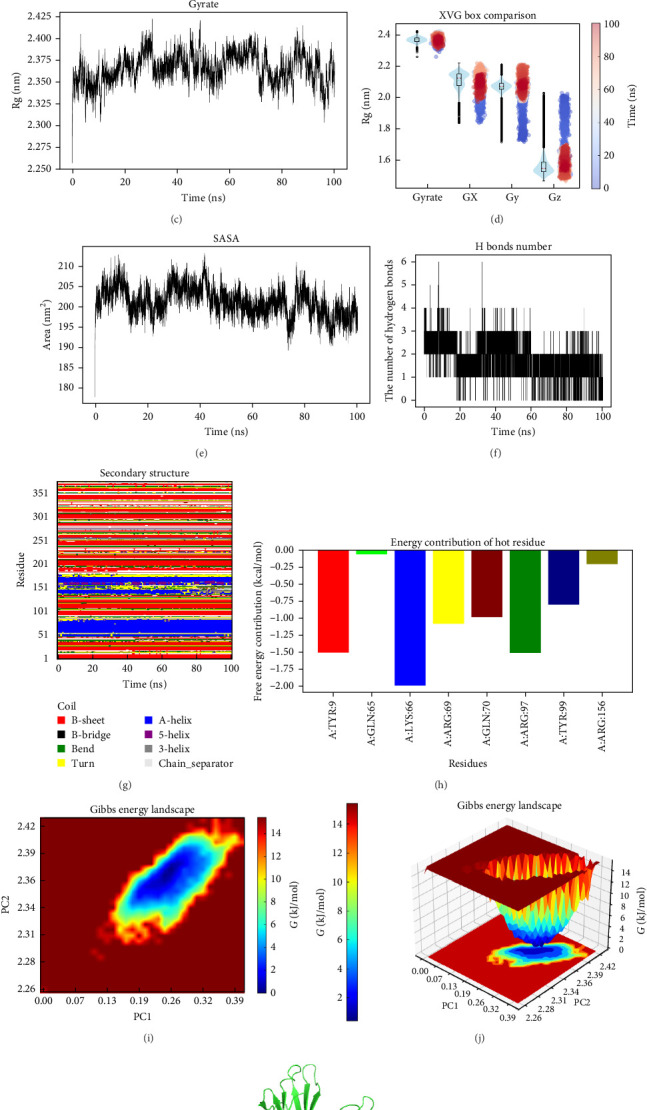
Molecular dynamics simulation analysis of the HLA-C–metronidazole complex. (A) RMSD curve of metronidazole (red line), HLA-C (black line), and the HLA-C–metronidazole complex (blue line). (B) RMSF curve of HLA-C. (C) Rg curve of HLA-C. (D) Boxplot of the Rg curve. (E) SASA of HLA-C–metronidazole. (F) Hydrogen bonds of the HLA-C–metronidazole complex. (G) The secondary structure evolution of HLA-C–metronidazole over time. (H) Energy contribution of hot residue of small molecules and proteins in the simulated 100 ns process. (I, J) Three-dimensional and two-dimensional Gibbs free energy landscape of the HLA-C–metronidazole complex. (K) Conformational analysis of HLA-C–metronidazole complex at the lowest Gibbs energy moment.

To further confirm the binding of Metronidazole with HLA-C, molecular dynamics simulations were conducted and the RMSD, RMSF, H-bonds, SASA, and Rg of the HLA-C–metronidazole complex were calculated. The changes in the HLA-C and metronidazole binding complex before and after molecular dynamics simulation are shown in Figure 9A. The RMSD curve of metronidazole (red line) remains stable at approximately 0.13 nm (Figure 9A). Additionally, the RMSD curve of HLA-C (black line) is stable between 0.24 and 0.27 nm from 20 to 100 ns. The RMSD curve of the HLA-C–metronidazole complex (blue line) remains around 0.28–0.32 nm from 20 to 100 ns, with slight fluctuations, indicating that the binding of metronidazole to HLA-C is relatively stable.

Residues 10–40, 140–160, 180–190, and 210–230 on Chain A of HLA-C show greater residual flexibility, while residues 10–40 on Chain B also exhibit similar characteristics (Figure 9B). The Rg value of HLA-C remains stable between 30 and 50 ns and 60 and 90 ns, with an average Rg value ranging from 2.375 to 2.400 nm (Figure 9C,D). The SASA value of HLA-C is larger before the binding with metronidazole and decreases after binding, indicating that metronidazole binding leads to a reduction in the surface area of HLA-C (Figure 9E). Simulation results show that the HLA-C–metronidazole complex has stable hydrogen bonds, with up to six hydrogen bonds formed (Figure 9F). The formation of hydrogen bonds between the ligand and receptor contributes to the stability of the complex. Further analysis of the secondary structure of HLA-C–metronidazole showed minimal changes in secondary structure over time, indicating excellent protein stability. In the HLA-C–metronidazole complex, the EMM values of Tyr9 and Lys66 are the highest, contributing most significantly to the residues (Figure 9G). The 3D and 2D Gibbs free energy profiles of the HLA-C–metronidazole complex show that when the Rg value is between 2.32 and 2.40 nm, and the RMSD value is between 0.13 and 0.35 nm, the Gibbs free energy of the complex is lower (Figure 9H–K). These results suggest that HLA-C is an important target for metronidazole.

To further investigate the binding stability of the HLA-C–metronidazole complex, the binding free energy of the HLA-C–metronidazole complex was calculated using the MM/PBSA method from the final 10 ns stable RMSD trajectory. As shown in Table 1, the total binding free energy of the HLA-C–metronidazole complex is −19.48 kJ/mol, with van der Waals energy of −18.14 kJ/mol, electrostatic energy of −19.20 kJ/mol, and gas phase energy of −37.34 kJ/mol, which favors the stability of the HLA-C–metronidazole complex system (Table 1).

## 4. Discussion

Periodontitis is a multifactorial disease influenced by both environmental and genetic factors [28, 29]. Increasing evidence indicates that individual susceptibility to periodontal tissue destruction varies among populations, primarily due to diverse immune and genetic factors [30–32]. Similar to other infectious diseases, HLA genes, which regulate antigen-specific T cell responses, are believed to be associated with the occurrence and progression of periodontitis [33]. Among these, the HLA Class I and Class II gene regions have been extensively studied [29]. Studies have revealed an association between HLA genes and genetic susceptibility to periodontal diseases [34]. Notably, significant differences in allele and haplotype frequencies of HLA Class II antigens have been observed, particularly in Iranian patient samples [35]. Numerous reports have highlighted the correlation between HLA Class I and Class II antigens and various types of periodontitis [36–38]. HLA-C, an important member of the major histocompatibility complex (MHC) Class I gene family, encodes a molecule that plays a crucial role in the immune system [39]. The high polymorphism of the HLA-C gene results in significant variability among individuals and populations. This diversity not only influences immune responses to pathogens but is also associated with susceptibility to various immune-related diseases. For example, specific HLA-C alleles have been shown to be associated with viral infections (e.g., HIV) [40], autoimmune diseases (e.g., psoriasis) [41], and transplant rejection.

This study combines DEG, WGCNA, immune cell infiltration assessment, scRNA-seq, and MR analysis to reveal the pivotal role of the HLA-C gene in periodontitis. The findings indicate that HLA-C not only exhibits a causal relationship with the onset of periodontitis but may also play a key role in immune regulation by modulating the activity of plasma cells and other immune cells, as well as their intercellular interactions. Initially, differential expression analysis of the GSE10334 dataset from the GEO database identified 167 DEGs, including 104 upregulated and 63 downregulated genes, laying the foundation for exploring the molecular mechanisms of periodontitis. Subsequent WGCNA revealed that among the 14 identified modules, the blue module exhibited the strongest correlation with periodontitis. GO and KEGG enrichment analyses further highlighted that the core genes associated with periodontitis are closely related to immune responses and cell signaling pathways, suggesting that immune activation and interactions among immune cells are critical factors in the pathological development of periodontitis. Immune cell infiltration analysis using the CIBERSORT algorithm revealed a significant increase in plasma cell infiltration in the periodontitis group, which aligns with the close relationship between immune responses and gene expression.

MR analysis further confirmed the causal relationship between HLA-C and periodontitis. SNPs in HLA-C were significantly and positively associated with the risk of periodontitis, suggesting that HLA-C may serve as a genetic susceptibility factor for the disease. This finding aligns with previous research on the role of HLA genes in immune-related diseases and further underscores the importance of HLA-C in periodontitis. Notably, HLA-C was found to correlate significantly with immune cell infiltration, including a positive correlation with plasma cells (*r* = 0.59) and negative correlations with resting DCs (*r* = −0.50) and CD8^+^ T cells (*r* = −0.53). These results suggest that HLA-C may influence the immune response in periodontitis by regulating immune cell infiltration patterns. scRNA-seq analysis revealed a marked increase in B cell activity in periodontitis patients, with a particularly elevated proportion of plasma cells, indicating that plasma cells may play a critical role in the pathological processes of periodontitis. Cell–cell communication analysis further demonstrated that plasma cells in the HLA-C-positive group exhibited more frequent and stronger interactions with other immune cells, suggesting that HLA-C may participate in the immune response of periodontitis by regulating interactions between plasma cells and other immune cells.In terms of drug prediction, analysis based on the DSigDB database identified metronidazole as the most promising HLA-C-associated therapeutic agent. MD analysis revealed a strong binding affinity between metronidazole and HLA-C, suggesting its potential as a therapeutic drug and providing new directions for the treatment of periodontitis.

Molecular dynamics simulation is a powerful computational method employed to examine the dynamic interactions between targets and small molecules, offering valuable insights into their mechanisms of action and aiding in drug design [42]. To further validate the stability of the complex and provide theoretical support for its potential biological function, we conducted a 100 ns MD simulation analysis of the HLA-C–metronidazole complex system. The RMSD results indicated that their binding was relatively stable. The RMSF analysis revealed significant structural domain flexibility between HLA-C and metronidazole. The smaller Rg suggested that the structure became more compact after binding. The SASA analysis showed a reduction in the surface area upon binding and many hydrogen bonds were formed between the complexes. The Gibbs free energy calculation results demonstrated that both complexes formed a single, sharp lowest energy region, indicating that the binding resulted in the most stable and compact state. Overall, the molecular dynamics simulation results suggest that the HLA-C–metronidazole interaction exhibits good stability in terms of the overall protein structure, internal stability, compactness, surface features, and interaction strength, providing reliable theoretical support for predicting its biological function.

In conclusion, this study highlights the critical role of HLA-C in the immune response of periodontitis, particularly in immune cell infiltration and intercellular interactions. It offers new insights into the molecular mechanisms of periodontitis and provides a theoretical basis for the development of HLA-C-targeted therapeutic agents. While our computational results suggest a strong binding affinity between metronidazole and HLA-C, we acknowledge that experimental validation is essential to confirm the therapeutic relevance of this interaction. Since the current study is based solely on computational predictions, we recognize the limitation arising from the absence of in vitro or in vivo functional validation. To address this, future studies will incorporate surface plasmon resonance (SPR) assays to assess the binding stability of HLA-C with metronidazole in vitro. Additionally, cellular experiments will be conducted to evaluate the functional impact of HLA-C–metronidazole interactions on immune cell activity and periodontitis progression. These experimental validations are crucial for further supporting the potential use of metronidazole as a therapeutic agent in periodontitis.

## Figures and Tables

**Table 1:** Binding free energy analysis of HLA–metronidazole complexes (kcal/mol).

Contribution components	HLA–metronidazole
Δ_VDWAALS_	−18.14 ± 0.29
Δ*E*_elec_	−19.20 ± 0.91
Δ*E*_GB_	20.53 ± 0.02
Δ*E*_surf_	−2.67 ± 0.02
Δ*G*_gas_	−37.34 ± 0.95
Δ*G*_solvation_	17.87 ± 0.03
ΔTotal	−19.48 ± 0.95

## Data Availability

The data can be requested from the corresponding author.

## Supporting Information

Supporting Information 1 Supporting Information Table 1. Classical biomarkers and B cell classical biomarkers were used to annotate cell clusters after clustering.

Supporting Information 2 Supporting Information Table 2. To further validate the accuracy of the annotations, a comparison was made with the PanglaoDB database, a comprehensive resource for human and mouse single-cell RNA-seq data.
